# Evaluation of the Safety of Percutaneous Dilational Tracheostomy Compared with Surgical Tracheostomy in the Intensive Care Unit

**DOI:** 10.1155/2019/2054846

**Published:** 2019-11-23

**Authors:** Yuta Suzuki, Takeshi Suzuki, Yuko Yamamoto, Ayano Teshigawara, Jun Okuda, Tomohiro Suhara, Tomomi Ueda, Hiromasa Nagata, Takashige Yamada, Hiroshi Morisaki

**Affiliations:** ^1^Department of Anesthesiology, Keio University School of Medicine, 35 Shinanomachi, Shinjuku-ku, Tokyo 160-8582, Japan; ^2^Department of Anesthesiology, Tokai University School of Medicine, 143 Shimokasuya, Isehara, Kanagawa 259-1193, Japan

## Abstract

**Background:**

Tracheostomy is a necessary procedure for patients who require long-term mechanical ventilation support. There are two methods for tracheostomy in current use: surgical tracheostomy (ST) and percutaneous dilational tracheostomy (PDT). In the current study, we retrospectively compared the safety of both procedures performed in our intensive care unit (ICU).

**Methods:**

In this study, we enrolled subjects who underwent tracheostomy in our ICU between January 2012 and March 2016. We excluded subjects who were <20 years old and underwent tracheostomy in the operating room. As a primary outcome, we evaluated the rate of complications between ST and PDT groups. The length of ICU stay, time to tracheostomy from intubation, and the rate of mechanical ventilation and mortality at 28 postoperative days were also examined as secondary outcomes.

**Results:**

Compared with the ST group, the rate of all complications was lower in the PDT group (13.4% vs. 38.8%, *p*=0.007). Although the rate of intraoperative complications did not differ between the two groups (3.8% vs. 8.1%, *p*=0.62), relative to the ST procedure, the PDT procedure was associated with fewer postoperative complications (34.6% vs. 9.6%, *p*=0.003). Among postoperative complications, accidental removal of the tracheostomy tube and an air leak from the tracheostomy fistula were less frequent in the PDT group than the ST group. Between the two groups, there were no significant differences in their secondary outcomes.

**Conclusion:**

This retrospective study indicates that relative to ST, PDT is a safer procedure to be performed in the ICU. Fewer postoperative complications following PDT might be attributed to the small skin incision made during this procedure.

## 1. Introduction

Tracheostomy is a standard procedure followed in the intensive care unit (ICU) for patients who require long-term mechanical ventilation. There are basically two approaches for performing tracheostomy. Surgical tracheostomy (ST) is the traditional method that secures a tracheal fistula by placing a tracheal tube. ST is occasionally followed by some complications, including major bleeding, surgical site infections, and tracheal stenosis. The alternative method called percutaneous dilational tracheostomy (PDT) was first introduced by Ciaglia et al. in 1985 [1]. In recent years, PDT has gained popularity due to its simplicity and safety, demonstrating its superiority over ST. Although some previous studies have shown that PDT is associated with fewer complications (such as surgical site infection, major bleeding, stoma enlargement, tracheal tube dislodgement, and death) [2, 3], discrepancies have been observed among previous studies in this regard [4, 5].

This retrospective study aimed to evaluate the safety of PDT by comparing the rate of perioperative complications between PDT and ST procedures performed in our ICU.

## 2. Methods

### 2.1. Subjects and Their Selection

Before initiating the study, we received approval from the ethics committee of the Keio University School of Medicine (registration number: 20150078). Subjects who underwent tracheostomy in our ICU between January 2012 and March 2016 were enrolled in this study. Subjects were excluded from participating in the study if they were under 20 years of age and had undergone tracheostomy in the operating room. The need of informed consent was waived because of the retrospective design of this study.

### 2.2. Tracheostomy Procedure in Our ICU

For all subjects who required long-term mechanical ventilation support, the timing of tracheostomy was determined after discussion between intensivists and attending physicians. Usually, both medical and surgical subjects, who are expected to need a mechanical ventilatory support for more than two weeks, undergo tracheostomy. In subjects with a worse conscious level (JCS 100–300), tracheostomy is performed after one-week mechanical ventilation management. In our ICU, PDT is performed on medical patients by the intensivists who carry out multiple graduated dilator techniques using a PDT kit (Neo Perc, Medtronic, USA) at the bedside. Briefly, about 1.5 cm skin incision is performed at the first step, and a needle, guidewire, dilator, and tracheal tube are inserted into the lumen of the trachea with real-time bronchoscopy assistance under EtCO_2_ monitoring. On the other hand, attending physicians perform ST on surgical ICU patients, including trauma patients. Tracheostomy was performed by the senior resident supported by the instructor in both PDT and ST groups. One more physician was in charge of anesthetic management in both groups and bronchoscopy assistance in the PDT group. For difficult cases, including patients after esophageal cancer surgery, with coagulation disorder (platelet count less than 10 × 10^4^/*μ*L or PT-INR more than 1.5 or APTT more than 50 seconds), and those with a history of neck operation, ST is performed by the otolaryngologist in the operating room.

In the ICU, all tracheostomy procedures were performed under general anesthesia with propofol, fentanyl or buprenorphine, and muscle relaxant administration. Further, the tracheostomy tube was inserted between the 1^st^ and 3^rd^ tracheal rings and fixed with 2 sutures after the tracheostomy procedure.

### 2.3. Measurements and Outcomes

As baseline characteristics, data were collected regarding the following variables: sex, age, height, body weight, diagnosis, and sequential organ failure assessment (SOFA) score at ICU admission, method of tracheostomy (PDT or ST), and duration of invasive mechanical ventilation and blood analysis (hemoglobin level, platelet count, activated partial thromboplastin time (APTT), and international normalized ratio of prothrombin time) before tracheostomy.

As a primary outcome, we compared the rates of perioperative complications occurring during the tracheostomy procedure and until 14 postoperative days (POD) in PDT and ST groups; these complications including hypoxemia (arterial oxygen saturation < 90%) during the tracheostomy procedure, active bleeding which required some treatment such as blood transfusion and ligation of blood vessels, extrathoracic insertion of tracheostomy tube, reintubation during the procedure, subcutaneous emphysema, and pneumothorax were compared as an intraoperative complications; bleeding which needed some treatments such as suture, electrocoagulation, blood transfusion, and frequent change of gauze, accidental decannulation of tracheostomy tube, and air leak from the tracheostomy fistula were evaluated as an immediate postoperative complications, and pneumonia, granulation around the surgical site, and surgical site infection were also evaluated as a late postoperative complications. Furthermore, intraoperative complications and postoperative complications (immediate and late) were evaluated separately. As secondary outcomes, we also examined the length of ICU stay, time to tracheostomy from intubation, the duration of mechanical ventilation after tracheostomy procedure, and the rates of mechanical ventilation and mortality at 28 POD.

### 2.4. Statistical Analysis

Results are presented as mean ± standard deviation (SD) for the variables with normal distribution or as median (interquartile range) for those with nonnormal distribution. Comparisons between two groups were performed using Student's *t*-test or Mann–Whitney's *U* test, as appropriate. Categorical variables were compared with the Chi-squared test. A *p* value of <0.05 was considered statistically significant.

## 3. Results

This retrospective study was performed in a 10-bed ICU of Keio University Hospital, which is a 944-bed teaching hospital. Intensivists (anesthesiologists) treated all patients in cooperation with the attending physicians and medical specialists. While intensivists with no experience of ST performed tracheostomy using the PDT technique on medical ICU subjects, ST was mostly carried out by attending physicians on surgical ICU subjects. In challenging cases involving subjects with coagulopathy, neck stiffness, and those with neck, esophageal, and cardiovascular surgeries, otolaryngologists performed ST in the operating room.

During the study period (between January 2012 and March 2016), 101 subjects underwent tracheostomy at the bedside in our ICU. Among these, PDT and ST were performed on 52 patients in the PDT group and 49 patients in the ST group, respectively. No PDT procedure was converted to ST. As shown in Table 1, PDT and ST groups showed no significant differences in their baseline characteristics other than APTT (PDT vs. ST: 34.3 (29.0–41.7) vs. 31.8 (26.0–37.3) seconds, *p*=0.044). Diagnosis at ICU admission was significantly different between both groups (*p* < 0.001). While the number of respiratory failure patients who received PDT was higher than those receiving ST, ST procedure was performed more frequently in subjects with neurological disorders and trauma subjects (Table 1).

The results of primary outcome are presented in Table 2. Perioperative complications occurred less frequently in the PDT group than the ST group (13.4% vs. 36.7%, *p*=0.013). One subject in the PDT group and two subjects in the ST group had two complications during the study period. Between the two groups, we observed no significant differences in the complications during the tracheostomy procedure (Table 2) (3.8% vs. 6.1%, *p*=0.946). In both groups, no active bleeding which required some treatment happened. Hemodynamic status was almost stable in both groups except for three subjects in the PDT group and four subjects in the ST group, which required vasopressors to increase blood pressure. Relative to the ST group, the PDT group was associated with fewer postoperative complications (Table 2) (34.6% vs. 9.6%, *p*=0.003). Among postoperative complications, accidental decannulation of the tracheostomy tube and air leak from the tracheostomy fistula occurred more frequently in the ST group than the PDT group. Although secondary outcomes, including the length of ICU stay, time to tracheostomy from intubation, and the rate of mechanical ventilation and mortality at 28 POD, did not differ significantly between both groups, the duration of mechanical ventilation after tracheostomy was significantly longer in the PDT group compared with the ST group (Table 3).

## 4. Discussion

In this retrospective study, we compared the rates of complications between PDT and ST groups and evaluated whether PDT can be performed safely at the bedside in the ICU. Consistent with the previous reports [6], the rate of complications in the PDT group was lower than the ST group (13.4% vs. 38.8%, *p*=0.007). Our results indicate that PDT performed by intensivists at the bedside is a relatively safe procedure as the rate of complications associated with the PDT procedure was found to be very low (3.8%).

Previous studies have shown conflicting results regarding the safety of PDT procedures [2, 6, 7]. In a retrospective cohort study including 528 patients (367 PDT and 161 ST), Beltrame et al. reported that PDT was associated with fewer complications (such as hemorrhage, surgical site infection, and stoma enlargement), while cannula dislodgement occurred more frequently in patients who received the PDT procedure [6]. In a meta-analysis conducted by Putensen et al. the rate of complications was evaluated as a primary outcome in 14 randomized controlled trials including 973 patients. This study revealed that PDT techniques could be performed faster and had reduced rates of stoma inflammation and infection; however, they were associated with increased procedural difficulties [7]. Another review article, including 1,608 patients (813 in the PDT group and 795 in the ST group), showed that while PDT was superior to ST based on the infection rate and operative time, both techniques did not differ significantly in their rates of hemorrhage complications [2].

In our study, the two techniques did not demonstrate significant differences in the rates of all complications related to tracheostomy procedure, and postoperative major bleeding and surgical site infection. However, ST was associated with a more frequent occurrence of postoperative complications, such as accidental decannulation and air leak from the fistula. In the PDT group, the reduced rate of accidental decannulation and air leak from the fistula may be attributed to the small skin incision made during the PDT procedure for insertion of the tracheal tube. It is likely that a small incision (approximately 1.5 cm in diameter) fitted tightly with the tracheal tube could have prevented stoma problems, such as accidental decannulation and air leak from the fistula. However, there is no evidence that a small incision can prevent accidental decannulation of a tracheal tube, and one previous study demonstrated that PDT was associated with more frequent cannula dislodgment compared with ST [6]. Considering the retrospective design of this study, other factors associated with the different rates of these complications between two tracheostomy procedures should be examined prospectively in a future trial. The duration of mechanical ventilation after tracheostomy was significantly longer in the PDT group than that in the ST group, which could be attributable to the difference of characteristics between both groups where the majority in the PDT group was medical subjects.

The discrepancy between our study and previous reports could be attributed to some limitations that are discussed here. First, the sample size in our study was too small, compared with previous studies. Second, differences in patient characteristics may affect the rate of complications. In our study, the PDT group included more patients with respiratory failure or neurologic disorders, while ST was performed more frequently in patients after brain surgery and in those with traumatic brain or burn injuries. The rate of complications resulting from tracheostomy procedure may differ between surgical and medical ICU patients. Furthermore, the tracheostomies were performed by different physician groups: the intensivists for the PDT group, and the surgical attending for the ST group, which could cause the different rate of complications in both groups. However, considering that all procedures were performed by senior residents, and tracheostomy was performed in the operating room by the otolaryngologist for difficult cases, the effects of these differences could be attenuated. Third, the challenging cases with an anatomic abnormality or coagulation disorder were excluded in our study, and this could explain for the inconsistencies in the findings from our work and others. Fourth, other important outcomes, such as cost and tracheostomy procedure time, could not be collected from the electronic record. Finally, the follow-up duration to monitor postoperative complications was relatively short in the present study. Thus, it is possible that the rate of long-term complications, such as tracheal stenosis and granulation, may be different between both techniques.

Even though conflicting results were previously reported concerning the differences in the rate of complications between PDT and ST groups, most of the studies including ours have favored PDT over ST with regard to patient safety. One recent study has demonstrated that ultrasound-guided PDT is associated with similar complication rates, compared with bronchoscopy-guided PDT [8]. While we performed bronchoscopy-guided PDT in this study, we believe that the safety of PDT could be augmented by performing an ultrasound-mediated evaluation (before PDT) of the puncture site and locations of artery and vein.

## 5. Conclusion

Relative to ST, PDT can be performed by intensivists more safely at the bedside in the ICU. However, further study is warranted to validate our findings and assess the safety of PDT.

## Figures and Tables

**Table 1:** Characteristics of subjects in the PDT and ST groups.

	PDT group (*n* = 52)	ST group (*n* = 49)	*p* value
Age	71 (64–78)	65 (52–81)	0.24
Gender (male/female)	35/17	31/18	0.83
BMI	20.6 ± 3.7	21.6 ± 3.3	0.15
SOFA score at ICU admission	6 (4–8)	7 (6–9)	0.06
Laboratory data before tracheostomy			
Hemoglobin (g/dL)	10.2 ± 1.8	10.0 ± 1.8	0.46
Platelet (×10^4^/mm^3^)	21.0 ± 11.2	24.6 ± 12.8	0.14
PT-INR	1.08 (1.04–1.27)	1.06 (0.98–1.19)	0.22
APTT (seconds)	34.3 (29.0–41.7)	31.8 (26.0–37.3)	0.04
Reason for ICU admission, *N*			<0.001
Neurologic	17	33	
Respiratory failure	32	6	
Heart failure	2	1	
Sepsis	1	1	
Trauma	0	8	

**Table 2:** Primary outcomes.

	PDT group (*n* = 52)	ST group (*n* = 49)	*p* value
All complications, *N* (%)	7 (13.4)	18 (36.7)	0.013
Intraoperative complications, *N* (%)	2 (3.8)	3 (6.1)	0.946
Hypoxemia	0	1	
Active bleeding	0	0	
Extratracheal insertion	0	0	
Reintubation	0	0	
Subcutaneous emphysema	2	2	
Pneumothorax	0	0	
Postoperative complications, *N* (%)	5 (9.6)	17 (34.6)	0.003
Immediate postoperative complications			
Bleeding	4	6	
Accidental decannulation	0	5	
Air leak from the fistula	0	7	
Late postoperative complications			
Pneumonia	1	1	
Granulation of surgical site	1	0	
Surgical site infection	0	0	

**Table 3:** Secondary outcomes.

	PDT group (*n* = 52)	ST group (*n* = 49)	*p* value
Length of ICU stay (days)	14 (10–15)	12 (9–15)	0.42
Time to tracheostomy from intubation (days)	7.5 (6–11)	7.0 (5–10)	0.22
Duration of mechanical ventilation posttracheostomy (days)	20.5 (7–39)	7 (3–22)	0.005
Mechanical ventilation at 28 POD, *N* (%)	21 (40.4)	12 (24.5)	0.14
Survival at 28 POD, *N* (%)	46 (88.4)	46 (93.9)	0.55

POD: postoperative days.

## Data Availability

The data used to support the findings of this study are included within the manuscript.
